# Leadership styles and quiet quitting in school context: unveiling mobbing as a mediator

**DOI:** 10.3389/fpsyg.2025.1538444

**Published:** 2025-04-30

**Authors:** Hüseyin Ergen, Fahrettin Giliç, Asena Yücedağlar, Yusuf İnandi

**Affiliations:** ^1^Department of Educational Sciences, Faculty of Education, Mersin University, Mersin, Türkiye; ^2^Mersin Directorate of National Education, Mersin, Türkiye

**Keywords:** leadership styles, autocratic leadership, democratic leadership, mobbing, quiet quitting, teachers

## Abstract

**Introduction:**

Leadership and mobbing can be the prominent antecedents of quiet quitting behaviours of teachers, which is also linked with their well-being. This relational study examines whether mobbing has a mediating role between teachers’ perceived leadership style and quiet quitting.

**Methods:**

This study employed structural equation modelling to analyse the questionnaire data from 411 teachers working in public schools in Turkey. Confirmatory factor analysis was conducted to determine whether the factor structures of the scales used in the study constituted a valid model, and SEM-based mediation tests were conducted to determine the relationships between the variables.

**Results:**

Autocratic leadership has a positive association with mobbing and quiet quitting while democratic leadership has a negative correlation with them. The structural equation modelling revealed that the level of mobbing perceived by teachers has a partial mediating role between the leadership style of school administrators and teachers’ quiet quitting behaviours.

**Discussion:**

These findings have theoretical and practical implications for leadership studies, organizational behaviour and educational management. It contributes to educational leadership theories by demonstrating that autocratic and democratic leadership styles influence teacher engagement not only directly but also through workplace mobbing.

## 1 Introduction

In the classical management approaches, many psychological, sociological and other needs of people were ignored, in short, the employees were not valued, however, with the increase in the value given to people in modern management approaches, many organizational behaviors have become important (Argyris, 2017). The organization and individuals reaching their goals at the desired level is also closely related to these behaviors. If the organization’s efficiency is to be improved, it is necessary to take into account the interests and needs of the employees and arrangements should be made accordingly. When organizations make arrangements for the interests and needs of the employees, the job satisfaction, motivation, organizational commitment and performance of the employees increase, which makes it easier for the expectations of both the organization and the individual to be met (Darolia et al., 2010; Pevec, 2023).

However, this does not always happen at the desired level. Sometimes, serious problems can occur in organizations due to disruptions experienced by organizational managers and employees in their levels of self-actualization (Formica and Sfodera, 2022; Gopinath, 2020; Kaufman, 2023; Schoofs et al., 2022). While these problems cause a decrease in productivity for the organization and drive down the quality of product, they can also cause employees to experience distance from work, burnout and alienation, lower organizational commitment, job dissatisfaction (Alshmemri et al., 2017; Fairlie, 2011), and finally, quiet quitting (Harter, 2022). It is a common fact that one of the major antecedents of these experiences is the attitudes and behaviors of managers toward employees. The anxiety of not knowing what to do, especially with the pandemic, has caused managers to put unnecessary pressure on employees, and as this pressure gradually increases in intensity, employees sometimes end up quiet-quitting (Engelmann, 2022).

These pressures exerted by administrators have sometimes reached the level of mobbing, and have led to the prominence of mobbing and quiet quitting, especially in educational context. These two concepts, which are of critical importance in terms of the effectiveness and efficiency of educational institutions, directly affect the performance of teachers, job satisfaction and organizational commitment, as previously stated (Galanis et al., 2024). Given that schools are not only workplaces but also institutions that shape future generations, investigating their internal dynamics is crucial. In this context, leadership within schools plays a pivotal role in influencing both teacher satisfaction and student outcomes (Agyeman Mr and Aphane Ms, 2024). Besides, mobbing is also a critical issue in educational settings, affecting teachers’ mental health and job performance a study on workplace bullying in early childhood education settings highlighted the importance of a supportive and positive workplace culture, with strong leadership, in preventing negative organizational behaviors (McFarland et al., 2024). We can conclude that teachers who exhibit quiet quitting behaviors may deviate from the school’s ultimate goals, potentially impacting other institutions in society.

When the studies in the literature are examined, there are a limited number of findings revealing that the leadership characteristics of school administrators have a direct effect on both mobbing (Peker et al., 2018; Tura and Yardibi, 2018; Yağcı and Uluöz, 2017) and quiet quitting (Are, 2024; Memiş and Tabancalı, 2024; Tsemach and Barth, 2023). While Peker et al. (2018) found a positive association of autocratic leadership with mobbing and negative association of democratic leadership with mobbing, Yağcı and Uluöz (2017) revealed no significant link between transformational/transactional leadership and mobbing in educational context. On the other hand, Are (2024) addresses the higher instances of quiet quitting behaviors in schools where leader-member exchange is low. However, the number of comprehensive studies that address the dynamics of the relationships between these three variables and especially their reflections in educational institutions is quite low. Previous studies have highlighted the association between certain leadership styles and the prevalence of mobbing in organizations (Peker et al., 2018) and also explored the mediating role of mobbing in the relationship between leadership styles and various employee outcomes such as increased absenteeism and job dissatisfaction (Tsuno and Kawakami, 2015). Additionally, constructive leadership behaviors have been shown to reduce withdrawal intentions and behaviors among employees through the reduction of mobbing experiences (Ertureten et al., 2013). Despite these insights, there is a notable gap in the literature concerning the specific mediating role of mobbing in the relationship between leadership (autocratic/democratic) styles and quiet quitting behaviors within school contexts. Understanding how leadership styles may indirectly influence quiet quitting behaviors through the presence of mobbing is crucial for developing effective interventions to promote a healthier work environment and teacher wellbeing in schools. Results of this research may help to ensure that if the goals of educational institutions are achieved at a more desired level by considering these three variables *jointly*, teachers are happier and have higher job satisfaction, the expected behaviors from education will be realized more healthily.

### 1.1 Leadership

Although various leadership styles are suggested in management studies (Kahn et al., 2016; Seethalekshmi, 2021), these leadership styles can be broadly grouped as developmental and controlling approaches (Williams, 1999). The developmental approach fosters engagement, motivation, and wellbeing, whereas the controlling approach enforces discipline, consistency, and compliance. In educational settings, too much control can lead to teacher disengagement (quiet quitting). Understanding these approaches helps in selecting the right leadership style to reduce mobbing and enhance teacher commitment. In this study, democratic leadership from the developmental group and autocratic leadership style from the controlling group are discussed in relation to mobbing and quiet quitting since autocratic and democratic leadership styles represent two ends of the leadership spectrum, offering a clear contrast in decision-making processes and interpersonal relations within educational settings.

#### 1.1.1 Autocratic leadership

Autocratic leadership is usually associated with negative behaviors as being unethical, lack of empathy, leader self-worthiness and self-orientation, verbal hostility, use of power asymmetry and harming and belittling of others (Modliba and Treffers, 2025). They make employees do their jobs without giving them the right to choose, do not care about employees’ ideas or needs, and make decisions alone (Ferguson, 2011). This can lead to employees losing their sense of control over their own work and decreasing their intrinsic motivation. According to Ryan and Deci (2000) self-determination theory, the basis of employee motivation is constructed by autonomy, competence, and a sense of relatedness. Lack of autonomy under autocratic leadership can lead to employees not meeting these needs and, as a result, losing motivation. In addition, autocratic leaders do not give much importance to employees’ feedback or ideas. According to Breevaart and Bakker's (2018) studies on leadership and employee commitment, employees’ interactions with the leader and feedback exchange have a great impact on job satisfaction and commitment. When an autocratic leader blocks such feedback channels, employees feel worthless and their commitment to their jobs decreases. Employees who are under high performance expectations and constant pressure are also likely to experience burnout. In Maslach and Leiter's (2016) study on burnout and job satisfaction, it was stated that emotional exhaustion in the workplace leads to employees alienating themselves from their jobs. In summary, as stress and pressure increase under autocratic leadership, employees may be triggered to emotionally distance themselves from their jobs and develop “quiet quitting” behavior (De Hoogh and Den Hartog, 2008). Behavioral valence attributes constitute negative leadership behavior.

#### 1.1.2 Democratic leadership

Unlike autocratic leaders, democratic leaders try to ensure that the work is done by giving employees the right to choose, and they also take into account organizational communication and group dynamics, and they emphasize sharing and increasing willingness with mutual respect (Ferguson, 2011). In other words, democratic leaders try to create organizational power by providing an atmosphere where employees can share their feelings, ideas, and experiences, and by showing that they value everyone’s ideas (Brookfield, 2010). The way leaders communicate with employees can directly affect their motivation at work. Breevaart and Bakker (2018) argue that an effective leader can increase employees’ awareness of their work by frequently exchanging feedback with them, which can prevent quiet quitting.

### 1.2 Mobbing

Mobbing is another variable that is effective in the process where teachers, as education workers, develop quiet quitting behavior. Leymann (1996) defines mobbing as psychological violence that occurs through hostile and unethical communication, systematically applied by one or more people in the workplace toward another person or people. This psychological violence and pressure cause a process that leads to employees distancing themselves from work life. Uysal and Yavuz (2013) state that mobbing is more common in organizations with hierarchical and autocratic structures. The existence of mobbing in educational institutions has serious consequences not only at the individual level but also at the organizational level (Forrester, 2023). The study by Galanis et al. (2024) shows that 60% of educators exposed to mobbing experience serious psychological problems and 40% consider quitting their jobs.

### 1.3 Quiet quitting

One of consequences of mobbing is quiet quitting. The act of quitting, which began with a viral TikTok video in the summer of 2022, has become one of the most talked-about and popular topics related to workplaces (Liu-Lastres et al., 2024). Quiet quitting is explained as employees who lose their passion for their work, fulfilling their job responsibilities to a minimum level (Formica and Sfodera, 2022; Galanis et al., 2024) and mentally quitting their job silently (Lu et al., 2023). It refers to the process of employees psychologically detaching from their jobs before physically leaving their jobs. During this process, employees only fulfill the basic job requirements and avoid taking on additional responsibilities. Considered in this way, the concept of quiet quitting is not related to the individual leaving the job actually (Formica and Sfodera, 2022; Yıkılmaz, 2022) but is expressed as the individual performing his job with minimum effort and completing working hours with minimum performance in business processes. In quiet quitting, the employee does not worry about being efficient in the job, saves his personal energy and does not feel passion for the job. The desire for behaviors such as being successful in the job, being productive and showing superior performance is not much of the employee’s interest. The employee performs to the extent that he will not be fired from his job and fulfills the requirements of the job at a minimum level. For these reasons, Youthall (2022) described quiet quitting as a kind of rebellion against routine work life.

The leadership behaviors of school administrators are very important in prescribing whether teachers will experience quiet quitting. If school administrators behave particularly overruling, constantly threaten teachers, push them into uncertainty and put them in inextricable situations, teachers might not actually leave their jobs completely but will continue to work inefficiently by performing the behaviors required by quiet quitting.

### 1.4 Purpose and contribution of the study

The study investigates whether mobbing serves as a mediating factor between autocratic/democratic leadership styles and teachers’ tendencies toward quiet quitting. In line with this aim, the research provides valuable insights into how the leadership styles at the very ends of the spectrum can affect the work environment, specifically concerning the occurrence of mobbing and its subsequent impact on teacher engagement. Furthermore, findings can inform school administrators about the potential consequences of their leadership approach, emphasizing the importance of adopting styles that minimize negative behaviors like mobbing and promote a supportive work environment. By focusing on the mediating role of mobbing, this study contributes to a deeper understanding of the mechanisms through which leadership styles influence teacher behavior, particularly in the context of quiet quitting.

### 1.5 Model

**Figure 1:**
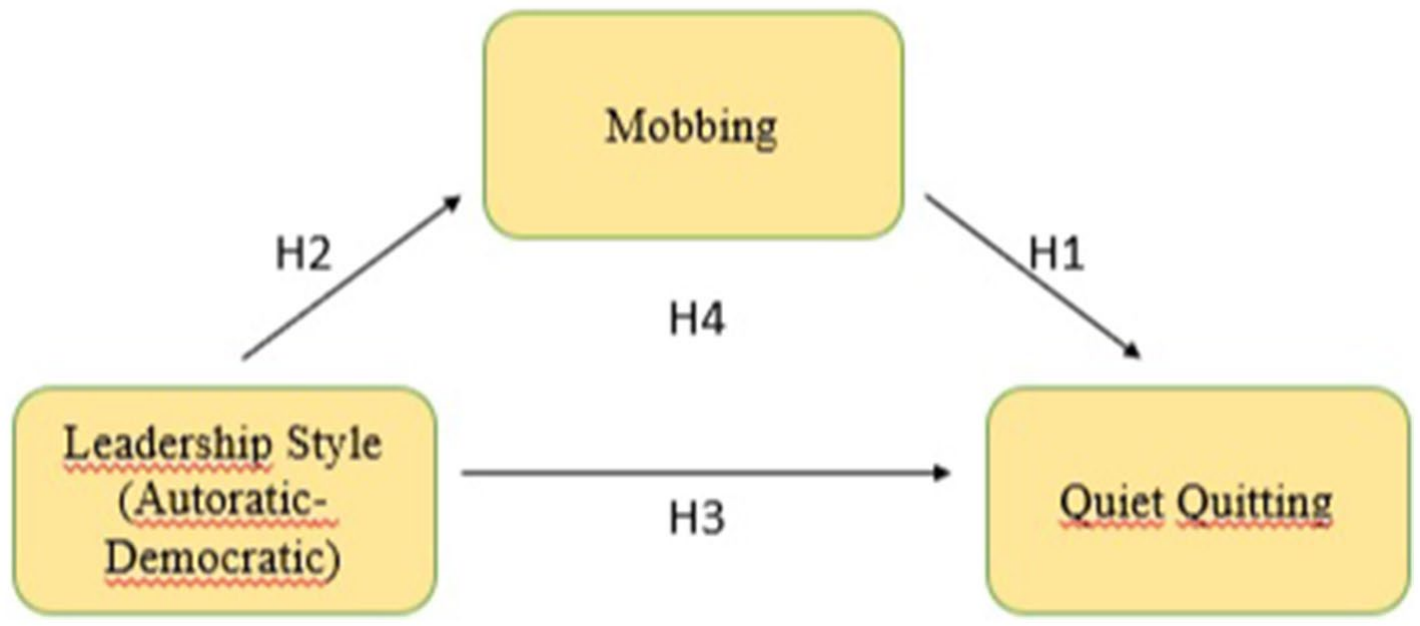
Hypothesized model of the study.

For the structural equation modeling study, a theoretical model was developed in which the relationships between the variables were examined in the literature. In this context, the studies conducted in the literature (Breevaart and Bakker, 2018; De Hoogh and Den Hartog, 2008; Galanis et al., 2024; Liu-Lastres et al., 2024; Lu et al., 2023; Maslach and Leiter, 2016; Serenko, 2023; Uysal and Yavuz, 2013) were taken as the basis in constructing the model. The theoretical model, which was created and tested to explain the mediating role of the mobbing perceived by teachers in the relationship between the leadership style of school administrators and teachers’ quiet quitting behaviors, which is the starting point of the research, is given in Figure 1.

Since a comprehensive relationship analysis has not been conducted among the variables mentioned in the literature review above, it is considered essential to study the direct or indirect relationships between the variables to be addressed in the research framework with structural equation modeling. It is also thought that it will contribute to the literature in terms of discovering possible causal relationships. Leadership style was considered as an important independent variable by the researchers in this study and was supported by the literature. It can be stated that the perceived leadership style is an important factor when the relationship between the level of mobbing perceived by teachers and their quiet quitting behaviors is considered. In other words, the research was conducted as an innovative study because it aimed to emphasize the importance of the mediating role of the mobbing between leadership style of school administrators and quiet quitting behaviors of teachers.

### 1.6 Research question and hypotheses

The purpose of this study is to examine whether perceived mobbing plays a mediating role in the relationship between school administrators’ leadership style and teachers’ quiet quitting behaviors. To investigate this relationship, a mediation analysis was conducted based on the framework illustrated in Figure 1. This study aims to explore and define both the direct and indirect relationships between the dependent and independent variables. The primary research question guiding this study is:

*“What is the mediating role of mobbing in the relationship between the leadership style of school administrators and teachers’ quiet quitting behaviors?”*

Existing literature highlights a positive association between mobbing and quiet quitting. Toxic workplace cultures, inadequate remuneration, and decreased employee wellbeing are key contributors to quiet quitting behavior (Nguyen, 2024). Similarly, a study by Arar et al. (2023) identifies mobbing and toxic organizational cultures as antecedents of quiet quitting. Based on the association between mobbing and quiet quitting, following hypothesis is proposed:

*Hypothesis 1*: There is a significant and positive relationship between mobbing and quiet quitting.

Within the relational chain between the variables of the study, leadership style serves as one of the initial influencing factors. Research suggests that autocratic leadership is positively associated with mobbing, while democratic leadership tends to mitigate it (Hoel et al., 2010; Peker et al., 2018). Furthermore, Hao et al. (2025) emphasize the importance of distributing leadership responsibilities to enhance employee resilience and prevent negative organizational behaviors such as quiet quitting. Based on this, following hypotheses are formulated:

*Hypothesis 2a*: There is a significant and positive relationship between autocratic leadership style and mobbing.

*Hypothesis 2b*: There is a significant and negative relationship between democratic leadership style and mobbing.

Autocratic leadership is characterized by centralized decision-making, strict control, and limited employee autonomy (Bass, 1990). Research suggests that such leadership styles contribute to increased workplace stress, reduced job satisfaction, and heightened perceptions of injustice (Den Hartog and De Hoogh, 2009). These factors can lead to employee disengagement, a core characteristic of quiet quitting, where employees mentally withdraw and perform only the bare minimum of their job requirements (Klotz and Bolino, 2022). Moreover, studies indicate that authoritarian leadership is positively correlated with emotional exhaustion and job dissatisfaction (Zhang et al., 2022), both of which are key antecedents of quiet quitting (Galanis et al., 2023; Hamouche et al., 2023). A rigid, control-heavy work environment discourages discretionary effort and fosters passive resistance (Miao et al., 2017), further reinforcing the association between autocratic leadership and quiet quitting. Democratic leadership, in contrast, emphasizes participatory decision-making, open communication, and employee empowerment (Goleman, 2017). This leadership style has been linked to higher levels of job satisfaction, organizational commitment, and intrinsic motivation (Avolio and Bass, 2004). Such conditions reduce the likelihood of quiet quitting by fostering a sense of belonging and engagement (Deci and Ryan, 2000). Research by Kim and Beehr (2020) found that democratic leadership significantly enhances psychological safety and job involvement, which are protective factors against disengagement behaviors like quiet quitting. Based on the literature above, the following hypotheses have been formulated regarding the relationship of democratic and autocratic leadership with quiet quitting.

*Hypothesis 3a*: There is a significant and positive relationship between autocratic leadership style and quiet quitting.

*Hypothesis 3b*: There is a significant and negative relationship between democratic leadership style and quiet quitting.

Autocratic leadership, characterized by centralized decision-making and limited employee input, has been linked to increased instances of workplace mobbing. A study investigating the relationship between leadership styles and mobbing among teachers found that autocratic leadership positively correlates with mobbing behaviors (Peker et al., 2018). Mobbing, in turn, leads to employee disengagement and withdrawal behaviors, which align with the concept of quiet quitting. Therefore, it is plausible that mobbing mediates the relationship between autocratic leadership and quiet quitting. Conversely, democratic leadership, which emphasizes participative decision-making and valuing employee input, has been associated with lower levels of mobbing. The same study on teachers indicated that democratic leadership negatively correlates with mobbing behaviors (Peker et al., 2018). Reduced mobbing under democratic leadership likely fosters a more positive work environment, decreasing the likelihood of quiet quitting. Thus, mobbing may serve as a mediating factor in the relationship between democratic leadership and quiet quitting. Existing literature suggests that mobbing can mediate the relationship between leadership styles and employee withdrawal behaviors. Autocratic leadership may increase mobbing, leading to quiet quitting, while democratic leadership may reduce mobbing, thereby decreasing the likelihood of quiet quitting. These insights provide empirical support for Hypotheses 4a and 4b.

*Hypothesis 4a*: Mobbing has a mediating effect in the relationship between autocratic leadership style and quiet quitting.

*Hypothesis 4b*: Mobbing has a mediating effect in the relationship between democratic leadership style and quiet quitting.

Direct studies on the mediation effect of mobbing between leadership styles and quiet quitting are limited. However, the relationships among these variables, as discussed, offer a theoretical basis for the proposed hypotheses.

## 2 Materials and methods

This is a relational research conducted to describe and explain the relationships between teachers’ perceived leadership style, mobbing and their quiet quitting behaviors. Relational research examines whether there is a relationship between two or more variables, and the researcher does not intervene in any way while these relationships are being addressed. In structural equation modeling, a causality claim is put forward without manipulation (Pedhazur and Pedhazur-Schmelkin, 1991). Although relational research is not cause-effect research, it provides clues about the cause and effect between the dependent and independent variables (Fraenkel and Wallen, 2011). The causality expressed in structural equation modeling is no more than theoretical causality (Hair et al., 1995; Tuijnman and Keeves, 1997).

### 2.1 Sample

The population consists of 13,381 teachers in public schools in central districts (Akdeniz, Toroslar, Yenisehir and Mezitli) of Mersin. There are 411 teachers (273 female, 66% and 138 male, 34%) in the sample formed by non-probability sampling. It refers to sampling techniques where not all members of the population have an equal chance of being selected (Levy and Lemeshow, 2007). According to a well-established sampling formula by Cochran (1977) for such larger populations, the recommended sample size is 372 for a 95% confidence level, meaning that a sample of 411 exceeds this recommendation and is statistically valid. Considering the demographics of the sample, the teachers consist of 75 (18.25%) from preschool, 104 (25.30%) from primary school, 146 (35.52%) from middle school, and 86 (20.93%) from high school. Among the teachers, 91 (22.14%) are single, while 320 (77.86%) are married. In terms of seniority, 25 (6.08%) have 1–5 years of experience, 69 (16.79%) have 6–10 years, 97 (23.60%) have 11–15 years, 90 (21.90%) have 16–20 years, and 130 (31.63%) have more than 21 years of experience.

The modeling study constructed with these collected data required testing the assumptions of multivariate statistical analyses since it was subjected to structural equation modeling (SEM) analyzes. One of the important assumptions that must be provided for SEM is the sample size. After the assumption tests, analyses were carried out with 403 observations. Considering the criteria regarding the minimum sample size in SEM-based studies, it was decided that the number of observations on which the analysis was carried out was sufficiently large and appropriate (Comrey and Lee, 1992; Kline, 2016; Tabachnick and Fidell, 2015).

### 2.2 Instruments

#### 2.2.1 Leadership style scale

The scale developed by Taş et al. (2007) is a five-point Likert type one and consists of 59 items. Cronbach’s alpha value for the entire scale was found to be 0.87. As a result of the factor analysis, it was stated that the scale consists of 5 dimensions: *(1) autocratic, (2) democratic, (3) laissez-faire, (4) transformational and (5) transactional leadership*. The autocratic (10 items) and democratic leadership (13 items) dimensions of the scale were used in the study. An example item is as follows: “Our school administrator provides employees with opportunities to express their creativity.” Reliability and validity findings related to the leadership dimensions used in this study are given in Table 1.

**Table 1:** Validity and reliability findings of measurement tools.

Dimensions	Cronbach alpha	CR	AVE	MSV	ASV	CR	Convergent validity	Divergent validity
Criteria	>0.70					>0.70	AVE > 0.50 <CR	MSV < AVE ASV < MSV
Autocratic leadership	0.81	0.90	0.50	0.57	0.27	✓	✓	✓
Democratic leadership	0.96	0.95	0.66	0.57	0.27	✓	✓	✓
Obstacles with work and career	0.91	0.91	0.58	0.43	0.29	✓	✓	✓
Work-related performance	0.87	0.88	0.52	0.41	0.21	✓	✓	✓
Indifference toward school	0.83	0.82	0.44	0.37	0.32	✓	✓	✓
Work-related depersonalization	0.55	0.69	0.43	0.41	0.21	✓	✓	✓

#### 2.2.2 Mobbing scale

The scale originally developed by Aiello et al. (2008) is a seven-point Likert-type scale consisting of 48 items and four dimensions. The adaptation of the scale to the Turkish language and culture was conducted by Laleoğlu and Özmete (2013). Since the Turkish adaptation of the scale was considered to be suitable for Turkish culture and language, it was adapted to be a five-point Likert-type scale by Laleoğlu and Özmete (2013). The Cronbach Alpha value of this scale was calculated as 0.94. As a result of the exploratory factor analysis in Turkish version, the scale consisted of five dimensions and 38 items. All factors in the scale explained approximately 73% of the structure. These dimensions were *(1) relationships with co-workers, (2) threats and harassment, (3) obstacles with work and career, (4) interference with private life, and (5) commitment to work*. The Cronbach Alpha coefficients of the five-factor scale structure were 0.96, 0.90, 0.90, special, 0.86, and 0.93, respectively, on the basis of sub-dimensions. High scores obtained from the scale indicate more exposure to mobbing, while low scores indicate less exposure to mobbing. The *obstacles with work and career dimension* (8 items) of the scale was used in the study. An example item is as follows: “I feel my career development is being deliberately hindered.” Reliability and validity findings related to this dimension used in this study are given in Table 1.

#### 2.2.3 Quiet quitting scale

The scale developed by Yücedağlar et al. (2024) is a five-point Likert type one and consists of 17 items. Factor analysis technique was used for the validity and reliability of the scale and the scale was examined under three factors. The total variance explained by the scale consisting of three factors as *(1) work-related performance, (2) indifference toward school and (3) work-related depersonalization* and 17 items is 58.64%. The Cronbach Alpha coefficients of the three-factor scale structure were calculated as 0.85, 0.83, 0.74, respectively, based on the sub-dimensions. It can be stated that as the score obtained from the scale increases, teachers exhibit more quiet quitting behaviors. An example item is as follows: “Doing less work than others would not be a problem for me.” The reliability and validity findings related to the quiet quitting scale used in this study are given in Table 1.

Confirmatory factor analysis (CFA) was conducted to determine whether the factor structures of the scales used in the study constituted a valid model. The aim of the CFA method is to discover the factor or factors by considering the relationships between the variables (Tabachnick et al., 2007) and to determine the sources of variance and covariance of the observed measurements (Jöreskog and Sörbom, 1993). Cronbach alpha (CA) and construct reliability values (CR) of the latent variables included in the study are also given in Table 1. Within the scope of validity findings, divergent and convergent validity evidence was also provided on the basis of measurement models. It is important to consider this evidence in detail to ensure the accuracy of the theoretical model created. To ensure convergent validity, the average variance extracted (AVE) values should be close to or above 0.50 (Fornell and Larcker, 1981). To ensure divergent validity, the Maximum Shared Squared Variance (MSV) and Average Shared Squared Variance (ASV) values should be calculated. The conditions required to ensure divergent validity are MSV ≤ AVE and ASV ≤ MSV (Yerlisu Lapa et al., 2020). Divergent validity is the condition that the expressions of the variables should be less related to factors other than the factor they belong to (Yaşlıoğlu, 2017). AVE, CR, MSV and ASV coefficients were used to test convergent and divergent validity. For convergent validity, AVE and CR values of the items were calculated and CR ≥ AVE ≥ 0.50 condition was required. If AVE values are < 0.50, CR ≥ 0.70 can be accepted as the criterion for convergent validity. For divergent validity, MSV ≤ AVE and ASV ≤ MSV conditions were required and the Cronbach alpha internal consistency coefficient of the scale and CR method were calculated (Gözmen and Aşçı, 2016). These relevant criteria and all calculated reliability and validity values are presented in Table 1.

When the reliability values in Table 1 are examined, the CA reliability coefficients for the sub-dimensions in the study were obtained as 0.81, 0.96, 0.89, 0.91, 0.87, 0.83, and 0.55, respectively. In Structural Equation Modeling and Confirmatory Factor Analysis studies, it is recommended to present the Construct Reliability (CR) value in addition to the Cronbach’s Alpha coefficient (Hair et al., 2009). The CR values calculated within the scope of Confirmatory Factor Analysis were evaluated by considering the criteria of Hair et al. (2009), and the values calculated for CR should be above 0.50. The CR values obtained from all sub-dimensions used in the study meet the relevant criteria. In the dimensions of indifference toward school and work-related depersonalization, which do not meet the AVE criteria, the CR values were calculated as 0.82 and 0.69, respectively. According to Fornell and Larcker (1981), if the CR value is higher than 0.60, it is acceptable for the AVE to be lower than 0.50. Based on these findings, it can be stated that these instruments provide reliable measurements for this study group.

### 2.3 Analyses

SPSS (version 20) and LISREL (version 8.7) were used in the analyses. Since the data were collected via Google Forms Electronic form before examining the assumptions, which is a prerequisite for the mediation study, there is no missing observation in the study. Each scale to be used in the mediation study was matched with the same sequence numbers and the assumptions of the multivariate statistics were examined. For normality test, skewness and kurtosis coefficients were examined and observed to be within the desired range (−1.5 to +1.5) (Tabachnick and Fidell, 2015). Accordingly, the skewness values are as follows: autocratic leadership (0.334), democratic leadership (−0.675), mobbing (1.016), and quiet quitting (1.105). The kurtosis values are as follows: autocratic leadership (−0.735), democratic leadership (−0.287), mobbing (0.460), and quiet quitting (1.273). Single (−4 ≥ z ≥ 4) and multiple outliers (by comparing Mahalanobis distances with the relevant degree of freedom and *p* < 0.001 table value) were examined (Hair et al., 1995; Tabachnick and Fidell, 2015), it was observed that the Z values varied between 4.90 and −2.60, thus one observation was a univariate outlier and seven observations (*χ*^2^_6_, 0.001 = 22.24) were not included in the analysis on the grounds of being multiple outliers.

In order to check the multicollinearity problem, which is another important assumption of multivariate statistics, the tolerance and Variance Inflation Factor (VIF) values were taken into consideration. When the literature reviews were taken into consideration (Tabachnick and Fidell, 2015), the tolerance values (0.360–0.512) for all items in the three scales were found to be above 0.20 and the VIF values (1.954–2.777) were found to be below 5. Another indicator of multicollinearity is the autocorrelation of errors, and this information was obtained by calculating the Durbin-Watson statistic. The calculated D-W statistic was calculated as 1.656 within the scope of the study. The values of the D-W statistic close to 2 indicate that the errors are independent (Kalayci, 2005). Based on these findings, no multicollinearity problem was encountered between the items of the measurement tools, which are the main points of the analysis.

Using path analysis instead of regression analysis to test the effect of the mediating role provides more reliable results (Meydan and Şeşen, 2011). In structural equation modeling, the cause-effect relationships of variables included in integrated hypotheses can be explained, theoretical models can be tested as a whole, and direct or indirect effects between variables can be determined (Kline, 2005; Raykov and Marcoulides, 2006). One of the important assumptions of SEM analysis is the testing of measurement models (Çokluk et al., 2016). In studies where CFA is performed, it is recommended to report RMSEA and 90% confidence interval, *χ*^2^/degree of freedom and significance value, CFI and SRMR values (Kline, 2016).

The suitability of the measurement models for the variables in the role of independent variable, dependent variable and mediator variable in the study, which is another prerequisite for mediation studies, is presented in Table 2. Model-data fit was evaluated within the framework of perfect fit and acceptable fit criteria in the relevant literature (Byrne, 1998; Hooper et al., 2008; Hu and Bentler, 1999; Kline, 2016).

**Table 2:** Measurement model results.

Variables	X^2^/df	RMSEA	SRMR	CFI	NFI	NNFI
CFA measurement model	3791.41/1074	0.079	0.079	0.97	0.95	0.97
Perfect-fit	≤ 3	≤0.05	≤0.05	≥0.95	≥0.95	≥0.95
Good-fit	≤3x^2^ / df ≤ 5	0.05 ≤ RMSEA ≤0.08	0.05 ≤ SRMR ≤ 0.10	0.90 ≤ CFI < 0.95	90 ≤ NFI < 0.95	90 ≤ NFI < 0.95

Regarding the model fit criteria in Table 2, it is seen that the tested measurement models match the perfect fit and good fit indicators. The dependent, independent and mediator variables in the study and the tested measurement model matched the perfect fit and good fit indicators, and accordingly, mediation tests were carried out.

In order to observe the mediation effect, three different relationships are predicted in the models created (Baron and Kenny, 1986, p. 116). These are the relationship between the independent variable and the dependent variable, the relationship between the independent variable and the mediator variable, and the relationship between the mediator variable and the dependent variable. In this relationship, when evaluating the evidence of mediation, the relationship between the independent variable and the mediator variable and the mediator variable and the dependent variable are taken as basis (McKinnon, 2008). In other words, in mediation studies, the indirect effects that are not visible at first in the relationship between the independent and dependent variables are examined. In the observed relationship between the dependent and independent variables, the mediator variable may show the entire relationship or only a part of it. In the case of full mediation, if the mediator variable is included in the analysis, the relationship between the dependent or independent variables is expected to become statistically insignificant, while in the case of partial mediation, the relationship between the independent variable and the dependent variable is expected to decrease (Burmaoğlu et al., 2013; McKinnon et al., 2010).

Before the analyses regarding the mediation studies, all the binary relationships between the variables to be considered within the scope of the model must be significant. In this context, the analyses of the binary relationships between the dependent, independent and mediator variables for which hypotheses were given for Model-1 and Model-2 included in the research were evaluated based on the measurement model outputs and presented in Tables 3, 4.

**Table 3:** Correlations between the variables.

Variables	Autocratic leadership	Mobbing	Quiet quitting
Autocratic leadership	1.00		
Mobbing	0.73^**^	1.00	
Quiet quitting	0.52^**^	0.58^**^	1.00

**p < 0.01.

**Table 4:** Correlations between the variables.

Variables	Democratic leadership	Mobbing	Quiet quitting
Democratic leadership	1.00		
Mobbing	−0.67^**^	1.00	
Quiet quitting	−0.46^**^	0.58^**^	1.00

**p < 0.01.

Based on these findings, the hypotheses H1, H2a, H2b, H3a and H3b, which were written in line with the theoretical model established in Figure 1 and, were confirmed and the prerequisites for the mediation study were provided.

## 3 Results

In this section, mediation analyses were performed step by step on two models whose theoretical frameworks were drawn. First, the direct relationship between the dependent and independent variables and the *t*-values that reveal the significance of this relationship are given, and secondly, the size of the relationship between the independent and dependent variable by adding the mediator variable to the models and the *t*-values calculated for this relationship are given.

### 3.1 Model-1

**Figure 2:**
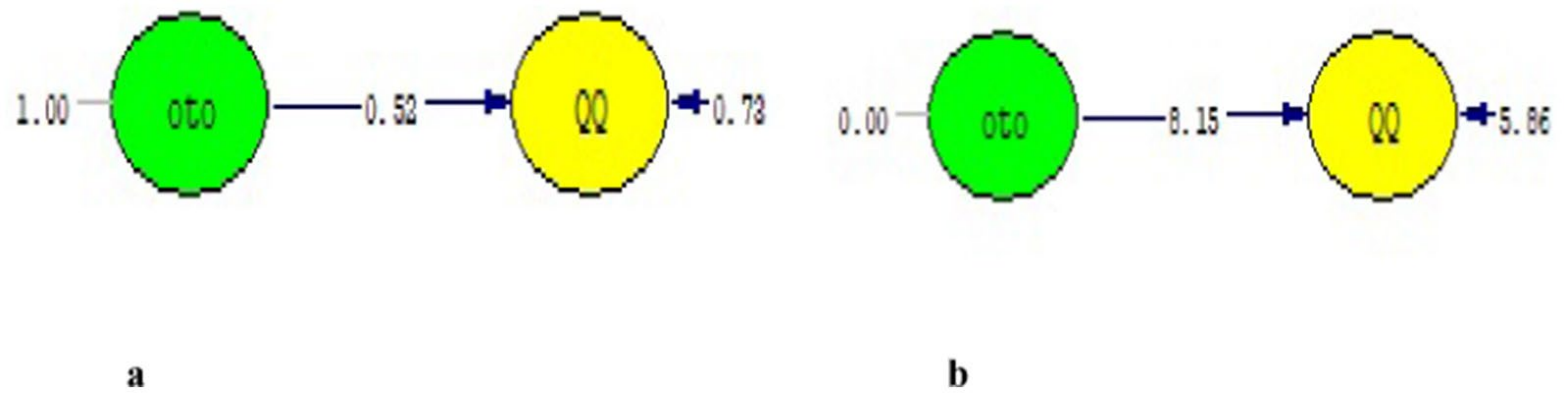
**(a)** Standard path coefficients. **(b)** *T*-value.

**Figure 3:**
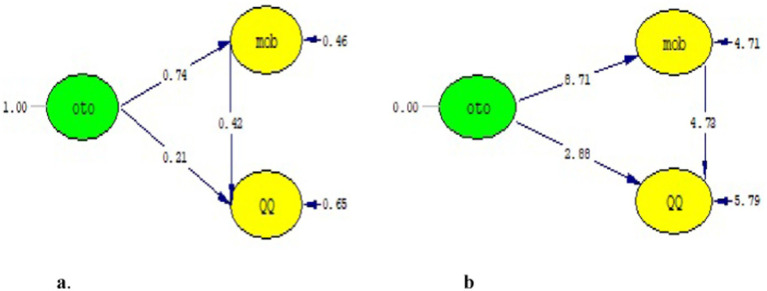
**(a)** Standard path coefficients. **(b)** *T*-value.

The model between the independent variable (autocratic leadership) and the dependent variable included in the mediation test is given in Figures 2a,b, and then the modeling including the mediator variable is given in Figures 3a,b. The results of the structural model created are given in Figure 2, and the goodness-of-fit findings related to the model are given in Table 5.

**Table 5:** Autocratic leadership and quiet quitting model fit values.

Model-Fit Category	X^2^/df	RMSEA	SRMR	CFI	NFI
Model	1598,59/323	0.099	0.082	0.94	0.92
Perfect-fit	≤ 3	≤0.05	≤0.05	≥0.95	≥0.95
Good-fit	≤3x^2^ / df ≤ 5	0.05 ≤ RMSEA ≤0.08	0.05 ≤ SRMR ≤ 0.10	0.90 ≤ CFI < 0.95	90 ≤ NFI < 0.95

It is seen in Figure 2a that the scores obtained from autocratic leadership have a significant effect on quiet quitting (*β* = 0.52, *p* < 0.01). The *t*-value in Figure 2b is also given as additional evidence for the significance of the relationship between them (*t* = 8.15, *p* < 0.01).

With regard to the goodness of fit criteria in Table 5, the fit values of the model established between the independent variable autocratic leadership and the dependent variable quiet quitting are within the criteria of perfect fit and acceptable fit. However, RMSEA, one of the statistical measures related to model error, produced values outside the criteria of fit in the literature. This value was not taken into consideration because it was stated that X^2^/df was not a good measure due to its dependence on sample size, that it could not be evaluated due to this structure, and that model fit could be decided based on other fit indices (Brown, 2015; Kline, 2016; Wheaton, 1987). As suggested in the relevant literature (Kline, 2016), the RMSEA value was interpreted together with the SRMR value and since other goodness of fit criteria also coincided with perfect fit and acceptable fit indicators, the measurement model for this latent variable was accepted as compatible with acceptable criteria, and mediation tests were carried out. In the second stage, the mediator variable “*mobbing*” was added to the model and Figures 3a,b were obtained.

It was concluded upon inclusion of mobbing in the model that the relationship between autocratic leadership and quiet quitting was relatively smaller compared to the relationship calculated in the first model (*β* = 0.21, *p* > 0.05). In addition, it was observed that the relationship from the independent variable to the mediator variable in the model was significantly high and positive (*β* = 0.74, *p* < 0.01) and the relationship between the mediator and dependent variable was significantly moderate and positive (*β* = 0.42, *p* < 0.01). The *t*-values calculated between the variables are given in Figure 3b.

After inclusion of the mediator variable in the model, the goodness of fit values were compared with the fit criteria, and it was revealed that the model had perfect-fit and acceptable indicators from a multiple evaluation perspective. When the goodness of fit measures in Table 6 and the values obtained from the structural model given in Figure 3 are taken into consideration, it is seen that the relationship between autocratic leadership and quiet quitting is partially explained by mobbing.

**Table 6:** Model-fit values of mobbing as mediator between autocratic leadership and quiet quitting.

Model-Fit Category	X^2^/df	RMSEA	SRMR	CFI	NFI
Model	2477,14/557	0.093	0.079	0.95	0.93
Perfect-fit	≤ 3	≤0.05	≤0.05	≥0.95	≥0.95
Good-fit	≤3x^2^ / df ≤ 5	0.05 ≤ RMSEA ≤0.08	0.05 ≤ SRMR ≤ 0.10	0.90 ≤ CFI < 0.95	90 ≤ NFI < 0.95

### 3.2 Model-2

**Figure 4:**
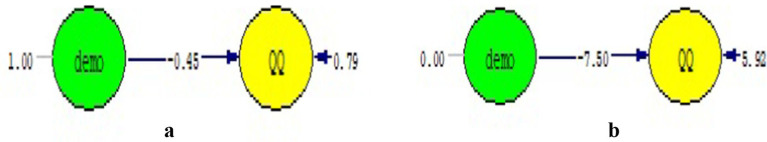
**(a)** Standard path coefficients. **(b)** *T*-value.

**Figure 5:**
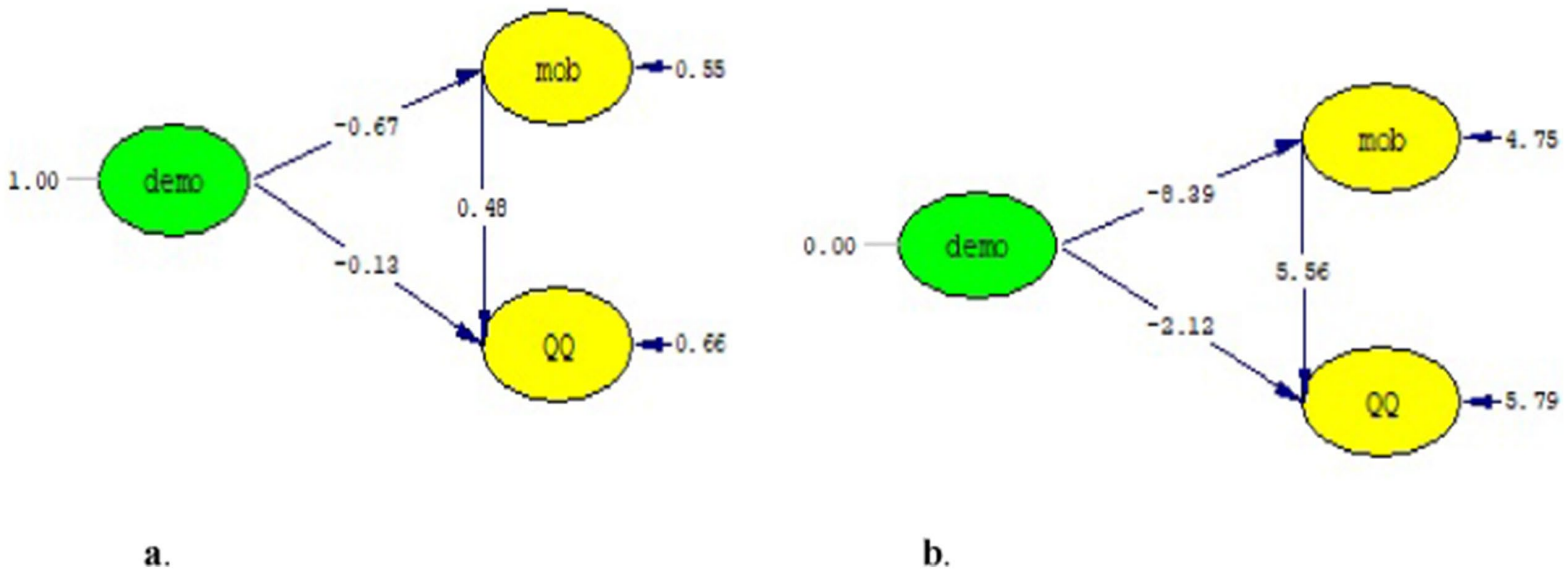
**(a)** Standard path coefficients. **(b)** *T*-value.

The model between the other independent variable (democratic leadership) and the dependent variable included in the mediation test is given in Figures 4a,b, then the model including the mediator variable is given in in Figures 5a,b. The results of the structural model created in Figure 4 and the goodness of fit findings related to the model are given in Table 7.

**Table 7:** Democratic leadership and quiet quitting model fit values.

Model-Fit Category	X^2^/df	RMSEA	SRMR	CFI	NFI
Model	1852,15/404	0.094	0.087	0.95	0.94
Perfect-fit	≤ 3	≤0.05	≤0.05	≥0.95	≥0.95
Good-fit	≤3x^2^ / df ≤ 5	0.05 ≤ RMSEA ≤0.08	0.05 ≤ SRMR ≤ 0.10	0.90 ≤ CFI < 0.95	90 ≤ NFI < 0.95

Figure 4a shows that democratic leadership have a significant and negative effect on quiet quitting (*β* = −0.45, *p* < 0.01). The *t*-value in Figure 4b is also given as additional evidence for the significance of the relationship between them (*t* = −7.50, *p* < 0.01).

When the goodness of fit criteria in Table 7 were examined from a multiple evaluation perspective, the fit values of the model established between the independent variable (democratic leadership) and the dependent variable (quiet quitting) were within the perfect fit and acceptable fit criteria. In the second stage, the mediator variable “mobbing” was added to the model and Figures 5a,b were created.

When mobbing was included in the model, the relationship between democratic leadership and quiet quitting was relatively smaller compared to the relationship calculated in the first model (*β* = −0.13, *p* > 0.05). In addition, it was observed that the relationship from the independent variable to the mediator variable in the model was significant, moderate and negative (*β* = −0.67, *p* < 0.01), and the relationship between the mediator variable and the dependent variable was significant, moderate and positive (*β* = 0.48, *p* < 0.01). The *t*-values calculated between the variables are given in Figure 5b. Baron and Kenny (1986) stated that mediation exists if the relationship between the independent variable and the dependent variable completely disappears or decreases when the mediator variable is included. The findings in Figure 5 denote that the expectation regarding the path coefficients is met in order to decide on the mediation effect. Considering the significance of the relevant relationships, mobbing can be stated to play a partial mediator role in the model. The goodness of fit values for this modeling are given in Table 8.

**Table 8:** Model-fit values of mobbing as mediator between democratic leadership and quiet quitting.

Model-Fit Category	X^2^/df	RMSEA	SRMR	CFI	NFI
Model	2797,87/662	0.090	0.082	0.96	0.94
Perfect-fit	≤ 3	≤0.05	≤0.05	≥0.95	≥0.95
Good-fit	≤3x^2^ / df ≤ 5	0.05 ≤ RMSEA ≤0.08	0.05 ≤ SRMR ≤ 0.10	0.90 ≤ CFI < 0.95	90 ≤ NFI < 0.95

After inclusion of the mediator variable in the model, the goodness of fit values were compared with the fit criteria and it was observed that the model had perfect-fit and acceptable indicators with the multi-perspective evaluation. When the goodness of fit measures in Table 8 and the values obtained from the structural model given in Figure 5 are taken into consideration, the relationship between democratic leadership and quiet quitting can be partially explained by mobbing.

Based on the findings above, the results of the hypotheses are presented in Table 9.

**Table 9:** Hypothesis testing results for leadership, mobbing, and quiet quitting.

Hypothesis	Statement	Statistical findings	Decision
H1	There is a significant and positive relationship between mobbing and quiet quitting.	r = 0.58, *p* < 0.01	Accepted
H2a	There is a significant and positive relationship between autocratic leadership style and mobbing.	r = 0.73, *p* < 0.01	Accepted
H2b	There is a significant and negative relationship between democratic leadership style and mobbing.	r = −0.67, *p* < 0.01	Accepted
H3a	There is a significant and positive relationship between autocratic leadership style and quiet quitting.	r = 0.52, *p* < 0.01	Accepted
H3b	There is a significant and negative relationship between democratic leadership style and quiet quitting.	r = −0.46, *p* < 0.01	Accepted
H4a	Mobbing has a mediating effect in the relationship between autocratic leadership style and quiet quitting.	Figure 2A, *β* = 0.52, *p* < 0.01 Figure 3A, *β* = 0.21, *p* < 0.01	Accepted
H4b	Mobbing has a mediating effect in the relationship between democratic leadership style and quiet quitting.	Figure 4A, *β* = −0.45, *p* < 0.01 Figure 5A, *β* = −0.13, *p* < 0.01	Accepted

## 4 Discussion and conclusion

This study examines the mediating role of mobbing in the relationship between the leadership styles (autocratic and democratic) of school principals and quiet quitting behaviors exhibited by teachers. The results of analysis show that there is a positive significant relationship of autocratic leadership with mobbing and quiet quitting, while they confirm the existence of a negative significant relationship of democratic leadership with mobbing and silent resignation. In other words, as school principals exhibit autocratic leadership behaviors, teachers may feel exposed to mobbing and tend to exhibit more quiet quitting behaviors. On the other hand, the democratic leadership style exhibited by school principals can be explained by the lower tendency of teachers to be exposed to mobbing and to quiet quitting. Some other studies in the literature support these results (Agervold, 2009; Blase and Blasé, 2002; Engelmann, 2022; Erdemir, 2023; Hoel et al., 2010; Liu-Lastres et al., 2024; Peker et al., 2018; Poussard and Çamuroğlu, 2009).

Autocratic leaders make decisions alone, do not give employees the right to choose, do not care about employees’ ideas or needs (Ferguson, 2011), try to maintain a rigid, hierarchical structure in the organization, and their priority is to get the job done rather than employees (Peker et al., 2018). In this leadership style, employees do not have a say, they experience a constant sense of pressure and fear, and they are not trusted, which can disrupt employees’ work-life balance. As the dose of autocratic leadership behaviors increases, the likelihood of employees being subjected to mobbing is higher (Agervold, 2009; Blase and Blasé, 2002; Poussard and Çamuroğlu, 2009; Uysal and Yavuz, 2013). Under autocratic leadership, employees lose their sense of control over their own work, which leads to a lack of autonomy and loss of motivation (Ryan and Deci, 2000), while closed or limited communication channels between leader and employee negatively affect employees’ organizational commitment and job satisfaction (Breevaart and Bakker, 2018). Therefore, it is very likely that employees who experience lack of motivation, low level of organizational commitment, job dissatisfaction, and burnout will tend to quit quietly (Formica and Sfodera, 2022; Forrester, 2023; Galanis et al., 2024; Hamouche et al., 2023; Liu-Lastres et al., 2024; Tsemach and Barth, 2023). In summary, while stress and pressure increase under autocratic leadership, employees may be triggered to emotionally distance themselves from work and develop “quiet quitting” behavior (De Hoogh and Den Hartog, 2008).

Democratic leaders, on the other hand, provide employees with autonomy in their work by giving them the right to participate in decisions and to choose (Ferguson, 2011). At the same time, they try to provide an environment where employees can share their feelings, experiences and ideas by valuing their ideas (Brookfield, 2010). Thus, employees have higher job satisfaction, organizational commitment, a sense of organizational citizenship and a more positive organizational culture (Peker et al., 2018). Due to these positive qualities of democratic leadership, it is anticipated that the effects of organizational factors related to mobbing will be reduced by creating constructive and functional working conditions (Houghton et al., 2021). In addition, while the communication style between employees as well as the leader and employee directly affects the motivation level of employees, it is suggested that the leader can increase the awareness of employees about their work by frequently exchanging feedback with them, and thus prevent silent resignation (Breevaart and Bakker, 2018). Individuals who feel happy in the organization they work for will be less likely to leave their jobs and experience burnout (İnandı and Büyükozan, 2013), alienation (İnandi et al., 2018) and cynicism (İnandi and Gılıç, 2021), which will positively affect educational organizations in every aspect.

In this study, a positive significant relationship between mobbing, which is considered as a mediator variable, and quiet quitting was revealed. Mobbing, defined as psychological violence to which employees are exposed (Leymann, 1996), is more common in organizations with hierarchical and autocratic structures, as explained above (Agervold, 2009; Blase and Blasé, 2002; Poussard and Çamuroğlu, 2009; Uysal and Yavuz, 2013). In the autocratic management approach, the priority given to work and performance rather than the ideas and needs of employees creates excessive workload on employees and can cause them to feel worthless. Employees who are under excessive workload (Galanis et al., 2024; Lawless, 2023; Lu et al., 2023) and feel worthless at work (Liu-Lastres et al., 2024; Serenko, 2023) are more likely to exhibit quiet quitting behavior. According to the research conducted by Galanis et al. (2024), 60% of educators exposed to mobbing experience serious psychological problems and 40% consider leaving their jobs, supporting this probability.

The results of analysis also revealed that mobbing is a partial mediator in the relationship between leadership style (autocratic and democratic) and quiet quitting. Erdemir (2023) states that autocratic leadership style triggers mobbing, but democratic leadership style helps prevent mobbing by establishing closer ties with employees due to its constructive nature. As stated in the abovementioned studies, mobbing is experienced most in organizations which are dominated by autocratic leadership behaviors. Namely, autocratic leaders prioritize work and performance and increase the pressure and workload on employees, thus paving the way for them to quietly quit. Autocratic leadership triggers quiet quitting largely through mobbing. Employees exposed to mobbing may exhibit quiet quitting behavior by emotionally disconnecting from work (Maslach and Leiter, 2016). On the other hand, as explained earlier, democratic leadership is a leadership style that encourages employees to participate in decision-making processes, values their ideas and opinions, prioritizes cooperation and communication, and helps increase employees’ motivation and job satisfaction, strengthen organizational commitment, and create a positive organizational climate. In such a work environment, mobbing behaviors can be expected to be less common. However, if mobbing is experienced in an organization despite democratic leadership, this may cause employees to quit quietly. In summary, while democratic leadership can be expected to prevent quiet quitting by reducing mobbing in general, the presence of mobbing can play a mediating role as a factor that undermines the positive effect of democratic leadership.

In conclusion, the study reveals a positive and significant relationship between autocratic leadership, mobbing and quiet quitting while democratic leadership has a significant but negative correlation with mobbing and quiet quitting. In addition, mobbing plays a partial mediating role between leadership styles (autocratic and democratic) and quiet quitting. Results of the study have theoretical and practical implications for leadership studies, organizational behavior, and educational management. The study contributes to educational leadership theories by demonstrating that autocratic and democratic leadership styles influence teacher engagement not only directly but also through workplace mobbing. The partial mediating role of mobbing suggests that negative experiences at work (such as bullying, exclusion, or mistreatment) partly explain why leadership style affects teacher disengagement. However, since the mediation is partial, other factors (such as work-life balance, burnout, or institutional policies) also play a role in quiet quitting. The study reinforces quiet quitting as an organizational, rather than purely personal, phenomenon. It aligns with Social Exchange Theory (SET), showing that when teachers perceive unfair treatment (mobbing), they reduce their efforts as a response. It also connects with Job Demands-Resources (JD-R) Theory, where mobbing acts as a job demand that depletes teachers’ energy, leading to disengagement. In practical terms, Administrators should avoid autocratic leadership practices (e.g., excessive control, lack of teacher autonomy) that contribute to mobbing and disengagement. Even democratic leadership needs reinforcement with anti-mobbing strategies to be fully effective. Training programs should emphasize fair communication, participative decision-making, and workplace respect. Lastly, Policymakers should recognize the link between leadership style, mobbing, and teacher retention when designing educational reforms.

### 4.1 Suggestions

This study emphasizes that leadership style alone does not determine teacher disengagement—the presence of mobbing is a critical factor. While autocratic leadership tends to increase mobbing and disengagement, even democratic leadership is not fully protective if mobbing is present. Based on these findings, it is recommended that school administrators should be given opportunity to learn the importance of democratic leadership in establishing teacher autonomy and creating a positive school climate, which will help prevent, or at least minimize, mobbing behaviors in educational contexts. To reduce quiet quitting and improve teacher retention, schools must train administrators to eliminate workplace bullying and establish policies ensuring fair treatment and respect for teachers.

### 4.2 Limitations

This study has several limitations in terms of generalizability, methodology and contextual factors. First, it was conducted only in Mersin, Türkiye, limiting the applicability of findings to other cities or countries. Next, the study only focuses on teachers, meaning its conclusions might not apply to other professions, such as healthcare, corporate settings, or public administration, where leadership-mobbing-quiet quitting dynamics may differ. Another limitation is about the design of the study. It is a cross-sectional study (data collected at one point in time), so it cannot establish causality, only associations between leadership, mobbing, and quiet quitting. To improve future research, scholars should;

- Expand the study to multiple cities to increase generalizability.
- Include other professions to see if results hold outside education.
- Use longitudinal methods to track changes over time.
- Consider additional variables (such as burnout, job satisfaction, school policies).

## Data Availability

The original contributions presented in the study are included in the article/supplementary material, further inquiries can be directed to the corresponding author.
